# Association between Genetic Variation in the *TAS2R38* Bitter Taste Receptor and Propylthiouracil Bitter Taste Thresholds among Adults Living in Japan Using the Modified 2AFC Procedure with the Quest Method

**DOI:** 10.3390/nu15102415

**Published:** 2023-05-22

**Authors:** Kyoko Aoki, Kanetaka Mori, Shohei Iijima, Masato Sakon, Nariaki Matsuura, Tsuneto Kobayashi, Masashi Takanashi, Takeshi Yoshimura, Norio Mori, Taiichi Katayama

**Affiliations:** 1 Department of Advanced Medical Treatment & Nutritional Science, United Graduate School of Child Development, Osaka University, Suita 565-0871, Osaka, Japan; mori-k00@cc.osaka-kyoiku.ac.jp (K.M.); matsuura@sahs.med.osaka-u.ac.jp (N.M.); katayama@ugscd.osaka-u.ac.jp (T.K.); 2 Division of Math, Sciences, and Information Technology in Education, Osaka Kyoiku University, Kashiwara 582-8582, Osaka, Japan; 3 Osaka Prefectural Hospital Organization, Osaka International Cancer Institute, Chuo-ku 541-8567, Osaka, Japan; shohei.iijima@oici.jp (S.I.); sakon-ma@oici.jp (M.S.); 4 I&H Co., Ltd., Ashiya 659-0066, Hyogo, Japan; kobayashi_t@i-h-inc.co.jp (T.K.); takanashi_m@i-h-inc.co.jp (M.T.); 5 Department of Child Development and Molecular Brain Science, United Graduate School of Child Development, Osaka University, Suita 565-0871, Osaka, Japan; 6 Department of Psychiatry and Neurology, Fukude Nishi Hospital, Iwata 437-1216, Shizuoka, Japan

**Keywords:** TAS2R38, bitter taste, PROP, genetic polymorphisms, QUEST method, Bayesian procedure, two-alternative forced-choice (2AFC) procedure

## Abstract

Individual taste sensitivity influences food preferences, nutritional control, and health, and differs greatly between individuals. The purpose of this study was to establish a method of measuring and quantifying an individual’s taste sensitivity and to evaluate the relationship between taste variation and genetic polymorphisms in humans using agonist specificities of the bitter taste receptor gene, *TAS2R38*, with the bitter compound 6-n-propylthiouracil (PROP). We precisely detected the threshold of PROP bitter perception by conducting the modified two-alternative forced-choice (2AFC) procedure with the Bayesian staircase procedure of the QUEST method and examined genetic variation in *TAS2R38* in a Japanese population. There were significant differences in PROP threshold between the three *TAS2R38* genotype pairs for 79 subjects: PAV/PAV vs AVI/AVI, *p* < 0.001; PAV/AVI vs AVI/AVI, *p* < 0.001; and PAV/PAV vs PAV/AVI, *p* < 0.01. Our results quantified individual bitter perception as QUEST threshold values: the PROP bitter perception of individuals with the PAV/PAV or PAV/AVI genotypes was tens to fifty times more sensitive than that of an individual with the AVI/AVI genotype. Our analyses provide a basic model for the accurate estimation of taste thresholds using the modified 2AFC with the QUEST approach.

## 1. Introduction

**Figure 1:**
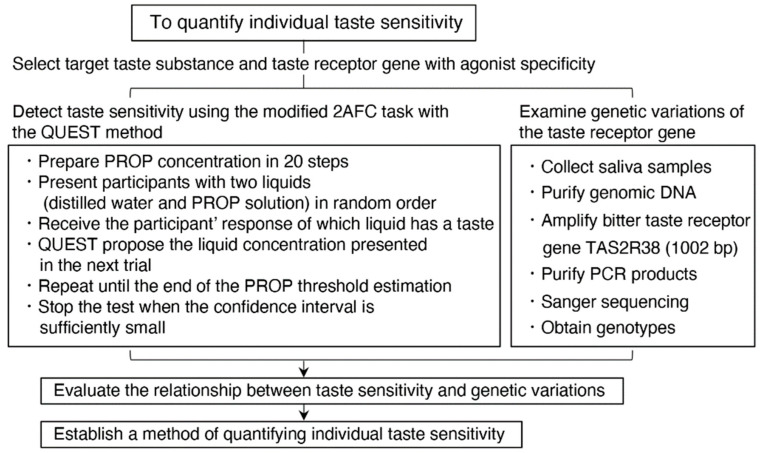
Summary of the protocol to quantify individual taste sensitivity in this study.

Individual taste sensitivity influences food preferences, nutritional control, and health. In mammals, there are five primary tastes: salt, sour, bitter, sweet, and umami (a taste of monosodium glutamate) [1,2,3]. Recently, genetic polymorphisms identified in taste receptors have been associated with differences in human sensitivity to bitter [4,5,6,7,8,9,10,11,12,13], sweet [14,15,16], umami [17,18,19], and salty [20] compounds. Therefore, when evaluating an individual taste perception, the genotype of taste receptors must first be analyzed and compared it with the results of taste sensitivity measurements. In the present study, we selected a target taste receptor gene and taste substance with agonist specificity so that the association could be simply understood. Taste sensitivity has been estimated in a variety of ways, as described later; however, a more precise and statistical method is required for the quantification of taste thresholds to evaluate the degree of inter-individual and intra-individual differences in taste sensitivity. The protocol for this study, which aimed to quantify individual taste sensitivity, is shown in Figure 1.

There are mainly two methods for the study of taste perceptions. One requires participants to answer questions of taste intensity, and the other requires participants to compare different concentrations of taste stimuli to obtain an objective measure of taste sensitivity. As an example of the first method, taste intensity scaling data have been collected using traditional Visual Analogue Scales (VAS) and general Labelled Magnitude Scales (gLMS), a semantically labeled line scale [21] anchored to the top 100 “strongest imaginable sensations of any kind” [22]. These scales require participants to indicate a subjective intensity on a rating line and, consequently, the measured taste sensitivities also become subjective values. These methods were often used for comparing the perceived strength of a taste stimulus and for suprathreshold measurements. The second method is a more objective method to measure a taste threshold for a taste stimulus, where the threshold indicates the minimum concentration of a substance detectable by the sense of taste. For example, the filter paper disk method requires participants to recognize thresholds for the four basic tastes on a paper disk on their tongue using five concentrations of test solutions [23] using taste-disc (Sanwa Kagaku Kenkyusho, Nagoya, Japan). However, the sets of infused disks have only five stepped concentrations and do not allow the detection of a quantitative value of an individual’s taste perception. The taste thresholds estimated by these previous methods are not only consecutive values, but also subjective values influenced by a participant’s own personal views or the order of tasting and the fixed concentration of the test solutions. The thresholds were assessed with more accurate methods in the last decades; for example, a variation in a three-alternative forced-choice (3AFC) and the ascending-concentration procedure [24]. The taste detection threshold (TDT) test employed a two-alternative forced-choice (2AFC), staircase, and tracking procedure, which reliably measured the taste detection thresholds for sweet, salty, and umami tastes from childhood to adulthood by controlling subjective response biases [25].

In this study, we used a modified 2AFC procedure with an adaptive staircase procedure based on Bayesian estimation of the QUEST method [26]. The use of the QUEST method in combination with the 2AFC procedure was mentioned in the original QUEST paper [26,27]. However, the QUEST method has been used with the yes-no task in taste threshold measurements [28,29,30] and has rarely been used with the 2AFC task. Our study is almost first to implement this combined method for the study of taste perception. Among threshold estimation methods, the forced-choice paradigm usually produces a criterion-free, unbiased threshold estimate [31]. In a typical 2AFC task, participants are presented with two stimuli simultaneously on each trial and are asked to identify the signal. In the present study, the 2AFC task is slightly modified; this 2AFC task requires participants to simply choose the liquid with a taste from two stimuli, one of which is a taste solution and the other distilled water. There has been a previous study using modified 2AFC with one set in taste solution and the other in distilled water as in our study [32]. For the modified 2AFC task, the answer given by the participant is simple and clear. It is “correct” or not and is, therefore, not influenced by the participant’s subjective viewpoint. Bayesian adaptive methods such as QUEST [26] produce estimates of psychometric function parameters more quickly than traditional staircase methods [28]. The QUEST method has been commonly used to measure sensory sensitivity in the visual field. Recently, QUEST has started to be used in the fields of gustatory [28,29,30] and olfactory sensitivity [33] and is still a new method in the field of the estimation of the taste thresholds. The QUEST method calculates a taste intensity every trial based on Bayesian estimation. After inputting the response of the participant in the first trial, QUEST updates the posterior probability density, estimates the current threshold of each trial, and proposes the concentration of the taste solution to be used in the next trial.

Among the five tastes, the bitter taste is the most suitable for investigating the association between taste sensitivity and genetic polymorphisms in taste receptors. Of the taste receptor genes for the five tastes, only the bitter taste receptor *TAS2R* genes do not contain introns that interrupt coding regions [34]. A single coding exon of each *TAS2R* is directly translated into protein; therefore, we need not consider the effects of polymorphisms in introns within the coding region. Moreover, the nucleotide diversity in *TAS2R* genes from geographically diverse samples is relatively high and results in many amino acid substitutions [35,36,37]. These properties of the *TAS2R* genes—no introns in their coding regions, and high nucleotide diversity—make it possible to evaluate the association between amino acid variation and phenotype by simply using genomic DNA to amplify the *TAS2R* genes.

Many genetic polymorphisms have been identified in the *TAS2R* genes, some of which cause functional changes in bitter-taste sensitivity. Whereas most TAS2R receptors have quite broad agonist spectra and respond to numerous chemicals, the *TAS2R38* receptor recognizes only one or a few compounds with thiouracil chemicals; for example, phenylthiocarbamide (PTC) or 6-n-propylthiouracil (PROP) [38,39,40]. The characteristic agonist specificities of *TAS2R38* provide a simple understanding of genetic polymorphisms and taste variation for bitter compounds of PTC or PROP. The significance of *TAS2R38* to bitter perception was first demonstrated by Kim et al. [8]; two common haplotypes were identified with profoundly different functional properties associated with the threshold detection of PTC. Most of the variability in PTC sensitivity can be accounted for by three single nucleotide polymorphisms (SNPs) at positions encoding amino acids 49, 262, and 296, which form 2 common haplotypes. The haplotypes of these polymorphisms encode “taster” PAV (Proline, Alanine, and Valine) and “non-taster” AVI (Alanine, Valine, and Isoleucine) forms of the receptor [8,41]. Moreover, PTC or PROP sensitivity is associated with heightened intensity of prototypical tastants—such as sucrose and quinine—even after statistically removing the contribution of the *TAS2R38* genotype [42,43,44]. Thus, bitter sensitivity to PTC or PROP and the *TAS2R38* genotype may be of use as a marker of whole oral sensation [44,45,46,47].

The purpose of this study was to establish a method of measuring and quantifying individual taste sensitivity by evaluating the relationship between taste variation and genetic polymorphisms in humans using agonist specificities of the bitter taste receptor gene, *TAS2R38*, with the bitter compound PROP. We detected the threshold of PROP bitter perception precisely using the modified 2AFC procedure with the QUEST method, examined genetic variation in *TAS2R38* in a population of adults living in Japan, and evaluated the genotype–phenotype association of bitter taste perception.

## 2. Materials and Methods

### 2.1. Sampling

First, a total of 118 unrelated healthy subjects from Japan were enrolled in the genetic analyses. They were given oral and written instructions to collect saliva samples half an hour or more after eating or drinking food. These saliva samples were kept at room temperature for DNA analysis. Informed consent was obtained from all participants. Second, 79 of the 118 participants who had time and cooperation for taste testing underwent PROP taste testing and filled out a general health questionnaire. As a result, association analysis between genotype and PROP bitter perception was performed for 79 subjects who underwent both tests (46 females and 33 males with an average age of 40.35 ± 12.17 years).

### 2.2. Stimuli and Apparatus

We used 6-propyl-2-thiouracil (PROP; Nacalai Tesque, Inc., Kyoto, Japan) to make tasting solutions. PROP was dissolved in distilled water (MilliQ). For the neutral stimulus, distilled water was used. Distilled water and solutions were kept in glass bottles. In the taste detection threshold test, 10 mL of these solutions were presented in plastic cups.

### 2.3. Taste Detection Threshold Test

**Figure 2:**
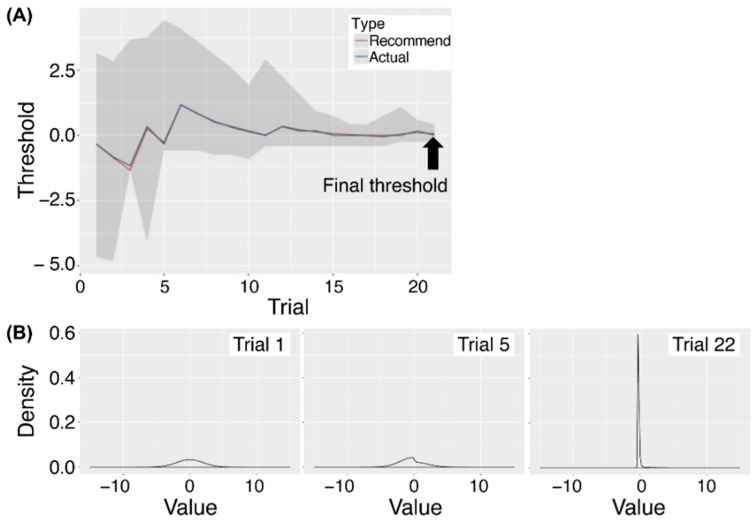
The procedure of the modified 2AFC with the QUEST method estimating the final threshold. (**A**) The transition of the thresholds converging to the final threshold using the QUEST method. The QUEST method calculates a taste intensity every trial based on Bayesian estimation. After inputting the response of the participant in the first trial, QUEST updates the posterior probability density, estimates the current threshold of each trial, and proposes the concentration of the taste solution to be used in the next trial. The rule for stopping the test was based on a 95% confidence interval indicated by the gray color when the interval was sufficiently small. On average, the QUEST method required 20 to 30 2AFC task trials. (**B**) The probability density function of trials 1, 5, and 22. Bayesian estimation needs the 2AFC tasks to continue until the shape of the posterior probability density shows sharpness and the threshold is sufficient for probability.

A summary of the protocol in this study is shown in Figure 1. PROP was used to measure the detection threshold of bitter taste. The PROP concentration varied in 20 steps (from 0.002 mM to 3.2 mM). The series of concentrations decreased by 1/6 in log steps. The concentration of PROP in the first trial was 0.015 mM (concentration step 6). The taste detection threshold was assessed using the modified 2AFC procedure with the QUEST method. In the 2AFC task used in this study, the participants received two liquids in turn, of which one was distilled water and the other was the PROP solution. They then had to choose which one evoked a taste. These two liquids were presented in a random order for each trial. The trial protocol was as follows: (1) participants rinsed their mouth with distilled water five times before the task; (2) they put the first liquid into their mouth and then spat it out; (3) they rinsed their mouth three times; (4) they received the second liquid, put it into their mouth, and spat it out; (5) they again rinsed their mouth three times; (6) they declared which liquid had a taste; and (7) the liquid concentration presented in the subsequent trial was proposed by the QUEST method. Based on the response to the previous trial, QUEST updated the posterior probability density and estimated the most plausible concentration of the threshold (Figure 2). At the next trial, we presented participants with liquid at the nearest concentration to the QUEST estimation. Steps (2) to (7) were repeated until the end of the PROP threshold estimation. The rule for stopping the test was based on a 95% confidence interval (Figure 2) when the interval between the upper and the lower limit after log transformation was lower than 0.6667 (i.e., the decline of one step × 4). On average, the QUEST method required 20 to 30 2AFC task trials about 30 min.

The presentation of a strong bitter taste could put a heavy strain on the participants; therefore, we established a restriction to avoid the presentation of much higher concentration of the bitter taste more than concentration step 12 (0.149 mM). If the next concentration proposed by QUEST was more than five steps from the previous trial, we modified the next concentration to be no more than three steps higher than that of the previous trial. This restriction reduced the possibility that participants would suddenly experience a strong bitter taste after having received weak solutions in previous trials.

### 2.4. Threshold Estimation

Following Watson and Pelli [26], the QUEST procedure that we used assumed a Weibull psychometric function that the slope parameter *β* is 3.5. The parameter *δ*, which represents an error rate regardless of the intensity of the stimulus, was set to 0.01. We defined the threshold intensity as the 75% correct point for the 2AFC. A formula that we used for calculating the best threshold intensity from the probability density function in the QUEST procedure was a mean of a posterior probability density function [27]. This method was proposed as the efficient and unbiased estimator, which is recommended in the Psychophysics Toolbox extensions [48].

The QUEST method is based on Bayesian estimation and estimates threshold values by incorporating prior knowledge about the parameters of the psychometric function and all of the participants’ response data. The QUEST procedure assumes a psychometric function that describes the relationship between the physical measure of a stimulus and the probability of a particular psychophysical response. Based on the assumptions, the threshold distribution is represented as a prior probability density function. This distribution is updated based on the tested intensity and the participant’s response on each trial. The optimal threshold estimation strength changes from trial to trial, and the latest threshold estimation is based on all previous responses of the participant. We wrote a program for estimating the threshold with the QUEST method in the MATLAB language with Psychophysics Toolbox extensions [48].

### 2.5. TAS2R Genotyping

Saliva samples were collected from all participants using Oragene DNA Self Collection kits (DNA Genotek, Ottawa, ON, Canada) and genomic DNA was purified according to the manufacturer’s protocol. The complete single coding exon of *TAS2R38* was amplified using the following polymerase chain reaction (PCR) primers: 5′-GCTTTGTGAGGAATCAGAGTTGT-3′ and 5′-GAACGTACATTTACCTTTCTGCACT-3′ [8]. Amplification of *TAS2R38* was performed as follows: 35 cycles of 10 s at 98 °C, 5 s at 55 °C, and 5 s at 72 °C in a total volume of 25 µL using PrimeSTAR Max DNA Polymerase (TaKaRa Bio, Kusatsu, Japan). The products were purified using ExoSAP-IT Express PCR Product Cleanup reagent (Thermo Fisher Scientific, Carlsbad, CA, USA) before Sanger sequencing by a commercial sequencing service. The obtained genotypes were aligned using Consed software [49]. The genotyping of 15% of samples were rerun to ensure reliability.

### 2.6. Statistical Analyses

We performed chi-squared test to assess the goodness-of-fit to Hardy–Weinberg proportions in our *TAS2R38* genotyping samples using the Genepop version 4.2 [50]. The threshold of the QUEST estimation was log-transformed before analysis. First, we conducted an analysis of covariance (ANCOVA) with the threshold as a dependent variable, the *TAS2R38* genotype as an independent variable, and age as the covariant. Second, we performed an analysis of variance (ANOVA), excluding age. We conducted the one-way ANOVA with *TAS2R38* as an independent variable and the threshold as a dependent variable. Furthermore, using multiple comparisons (Tukey’s HSD test), we investigated the significant difference between genotypes. These analyses were carried out in R Core team [51]. The ANCOVA was performed in the car package in R Core team [52] using type II sum of squares.

## 3. Results

### 3.1. TAS2R Polymorphism

Three SNPs—C145G, C785T, and A886G—were detected in the single coding exon of *TAS2R38* (1002 bp) from 118 unrelated Japanese individuals (Table 1). All three SNPs encoded amino acid substitutions at P49A in the first intracellular loop, A262V in the sixth transmembrane domain, and V296I in the seventh transmembrane domain. Rare variants are known to exist [6,8,36,53,54,55], but these were not found in our population. The genotype frequency distributions of *TAS2R38* were 31%, 53%, and 15% in all 118 genotyped samples for PAV/PAV, PAV/AVI, and AVI/AVI, respectively (Table 1), and were in Hardy–Weinberg equilibrium (*p* = 0.35). These genotype frequencies were similar for the 79 subjects who performed taste detection (Table 2) and were not different from those reported in Asian populations [55,56].

### 3.2. Relationship between the PROP Bitter Taste Threshold and the TAS2R38 Genotypes

**Figure 3:**
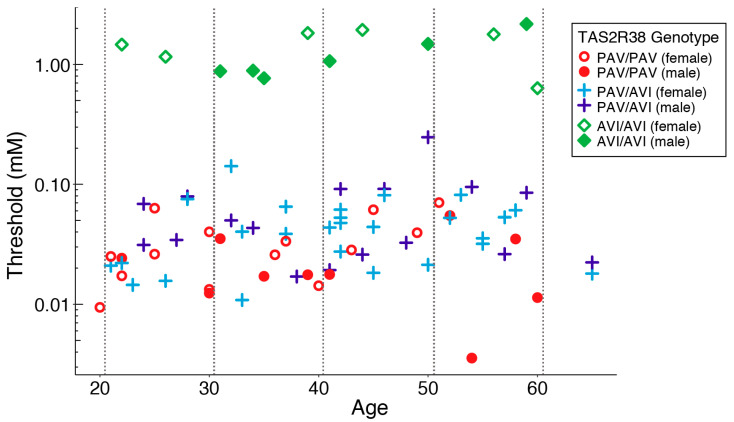
Scatter plot of PROP thresholds based on the *TAS2R38* genotype and age. The Y-axis is presented in a logarithmic scale.

**Figure 4:**
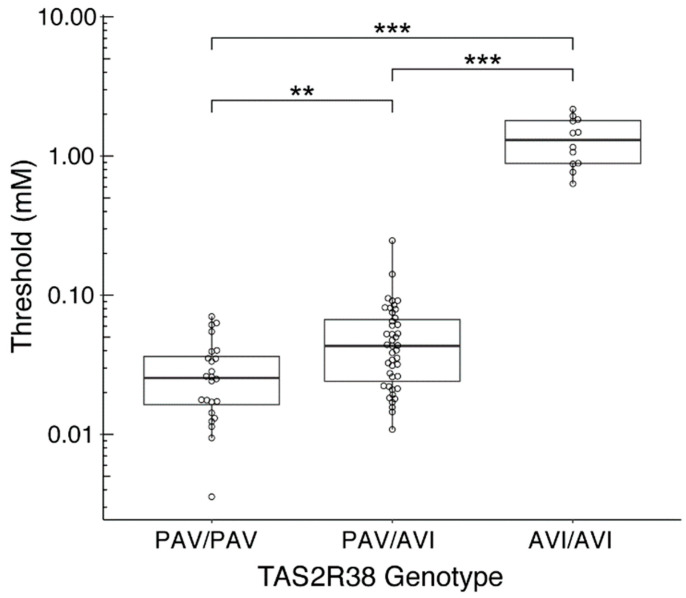
Boxplots of PROP thresholds for the *TAS2R38* genotype. Thresholds are different depending on the *TAS2R38* genotype. AVI/AVI exhibits significantly lowest sensitivity. There is also a significant difference between the higher sensitivity of PAV/PAV and PAV/AVI. The Y-axis is presented in a logarithmic scale. The lines inside the boxes indicate the median. Each individual threshold is shown by a circle. *** *p* < 0.001; ** *p* < 0.01.

Table 2 shows the characteristics of the participants in the PROP taste testing. Figure 3 shows a scatter plot of the threshold depending on the *TAS2R38* genotype and age. The ANCOVA indicated that the effect of the *TAS2R38* genotype was significant (*F*(2, 75) = 161.46, *MSE* = 12.790, *p* < 0.001, η^2^ = 0.81), while that of age was not significant (*F*(1, 75) = 1.01, *MSE* = 0.08). The one-way ANOVA with *TAS2R38* as an independent variable and the threshold as a dependent variable also revealed that the effect of *TAS2R38* was significant (*F*(2, 76) = 163.32, *MSE* = 12.940, *p* < 0.001, η^2^ = 0.81). The high value of η^2^ indicated that the *TAS2R38* genotype accounts for most of the PROP threshold variance. Multiple comparisons revealed significant differences between all genotype pairs: PAV/PAV vs AVI/AVI, *p* < 0.001; PAV/AVI vs AVI/AVI, *p* < 0.001; and PAV/PAV vs PAV/AVI, *p* < 0.01. To illustrate these analyses, Figure 4 shows a box plot with data presented by the *TAS2R38* genotype.

We also analyzed the data after removing the lowest value from the PAV/PAV dataset because of the possible influence of this outlier value on the statistical results. The ANOVA revealed a significant effect of the *TAS2R38* genotype (*F*(2, 75) = 176.94, *MSE* = 12.54, *p* < 0.001, η^2^ = 0.83). Multiple comparison still revealed a significant difference between PAV/PAV and PAV/AVI (*p* < 0.05). This analysis indicated that the difference between PAV/PAV and PAV/AVI was robust and did not depend on the lowest value in the PAV/PAV dataset.

## 4. Discussion

The present study demonstrates a clear relationship between the taste perception threshold of the bitter compound, PROP, and genetic variation in the bitter taste receptor gene, *TAS2R38*: the *TAS2R38* PAV homozygosity is associated with bitter sensitivity “taster”, while AVI homozygosity is associated with bitter insensitivity “non-taster”, and PAV/AVI heterozygosity represents a significantly lower sensitivity compared with PAV homozygosity (Figure 4). The thresholds for non-tasters with the AVI/AVI genotype could be separated from the threshold for others around 0.3 to 0.6 mM, that is, non-tasters could be separated only by the threshold values using the modified 2AFC procedure with the QUEST method without considering genotypes (Figure 4). The PROP bitter perception of individuals with the PAV/PAV and PAV/AVI genotypes was on average tens to fifty times more sensitive than that of non-tasters with the AVI/AVI genotype (Figure 4), and thus individual bitterness thresholds could be quantified and compared among individuals. This success can be attributed to first, for the modified 2AFC procedure used in this study, the answer provided by participants is a simple and clear “true or false” and is, therefore, not influenced by the participants’ subjective feeling of taste intensity. Second, the method requires 20 to 30 2AFC task trials for every participant. This is far more tasks than for the gLMS and paper filter methods, and also more than previous 2AFC task methods, which usually used five to ten trials. Third, the QUEST method can determine an individual’s final taste threshold as a continuous value without limiting the taste thresholds to the presented concentration of the taste stimulus. This allows us not only a relative determination of which person has the more sensitive taste perception, but also a numerical comparison of what degree the taste thresholds differed from each other. Fourth, the QUEST method calculates the posterior probability density and the threshold for every trial depending on the response of the participant and the 2AFC task is continued until the shape of the posterior probability density shows sharpness and the threshold is sufficient for probability (Figure 2). Therefore, the presented concentration of taste stimuli at each trial and the total number of the 2AFC tasks are not determined by the experimental organizer but by the response of the participant and the estimated final taste thresholds can be determined objectively. The QUEST method has been shown to have largely the same thresholds and higher reliability between measurements than other approaches to detect thresholds such as quick Yes−No or SIAM methods, all of which are used adaptive yes-no tasks instead of the 2AFC task [28,30]. 

In many previous studies, a clear significant difference in the taste threshold and the taste intensity has been described between PAV homozygotes and AVI homozygotes [5,7,8,13,41,42,43,53,57]. However, previous studies have shown varied results on whether PAV homozygotes and PAV/AVI heterozygotes differ significantly in taste threshold. There were several cases that showed significant bitter "taste intensity" differences between PAV/PAV and PAV/AVI during strong stimulation, especially at 3.2 mM PROP as measured by gLMS (e.g., [7,42,43,57]), but few cases (e.g., [8]) found significant "taste threshold" differences between PAV/PAV and PAV/AVI. One or two copies of the PAV allele are sufficient to shift the detection threshold from insensitive to sensitive [42]. The present study shows a significant difference of “taste threshold” between PAV homozygotes and PAV/AVI heterozygotes, as well as AVI homozygotes and PAV/AVI heterozygotes. The bitter sensitivity between the individuals with PAV/PAV and PAV/AVI could not be separated by taste threshold values alone but could be significantly differentiated by combining the results of *TAS2R38* genotypes. Both the *TAS2R38* genotypes and the modified 2AFC with the QUEST method enabled us to estimate precise taste threshold values and quantify individual bitter perception.

The potential and future directions of the method of quantifying an individual taste threshold are described below. First, the quantified bitter threshold to PTC/PROP may have utility as an individual phenotypic marker of whole oral sensation. Higher PTC/PROP sensitivity has been associated with a stronger perception of prototypic tastes such as sucrose and quinine [42,43,44], and such individual differences in bitter perception have influenced food preferences [58,59,60,61,62,63,64,65]. The polymorphisms in *TAS2R38* have been associated with increased alcohol intake [7,66], caffeine intake [67,68], food choice [69,70,71,72,73], and smoking behaviors [54,74]. Thus, the PTC/PROP threshold combining *TAS2R38* genotypes may enable prediction of individual taste sensitivity and food choice tendencies. Second, the individual bitter sensitivity to PTC/PROP will also be useful for clinical medical care for taste sensitivity changes or taste disorders for cancer patients. Recently, taste sensitivity measurement using QUEST have shown that when an additional rule is introduced to avoid repeated presentation of the same concentration over multiple consecutive trials, the taste threshold converges in a short time, approximately 14 trials and 6 min [28]. Further modifications to the QUEST setup to reduce time will allow future taste measurement in time-constrained situations, such as patients or children. In addition, for patients with taste disorders who still have difficulty measuring taste sensitivity, this study suggests that simply analyzing *TAS2R38* genotypes can predict the degree of an individual taste perception, which can be used to estimate whether taste sensitivity alteration or taste disorders will occur after medication, such as chemotherapy.

Limitations of the present study should be noted as follows. Our samples were primarily composed of adults living in Japan and genetic ancestry has not been estimated by genome-wide association studies; therefore, it is unclear whether these results may generalize to other groups. The present analyses only considered one gene, *TAS2R38*, and PROP bitter taste. The roles of other genes in PROP taste and of *TAS2R38* in tasting other compounds remain to be determined. We observed no significant difference between the bitter compound taste threshold and participant age, although previous study has reported a strong influence between age and PROP taste sensitivity [75]. Negri et al. [75] reported that children were more sensitive to PROP than adults, with the same *TAS2R38* haplotype also within mother-child dyads, and that the mother-child tasting differences decreased with age and was minimal when the children reached adolescence. The lack of a significant effect of age on PROP thresholds seen in our study suggests that the participants fell within a narrow age range, especially because there were no children. The relationship between the bitterness of concentrated PROP and fungiform papillae of the tongue differs across *TAS2R38* and the gustin polymorphisms [42,57,76]. Estimates of fungiform papillae number in Asian subjects may quantify their contribution to taste intensity. Since this study did not compare results using the QUEST method with other psychophysical tools or compare outputs with and without the QUEST method, it was not possible to show how much better the QUEST method is compared to other measurement methods. When the QUEST method is to be applied in clinical practice to quantify patients’ taste threshold, it needs to be improved to reduce the time required for threshold measurement.

In conclusion, the modified 2AFC procedure with the QUEST method enabled us to estimate precise taste threshold values and to evaluate the differences in taste sensitivity among individuals. Our analyses provide a basic model for accurate taste threshold estimation by analyzing the taste receptor gene and using the modified 2AFC procedure with the QUEST approach.

## Figures and Tables

**Table 1:** Details of SNPs observed in the human *TAS2R38* gene.

	Nucleotide			Amino Acid			No. of Samples	Frequency
Genotype	145	785	886	49	262	296		
PAV/PAV	CC	CC	GG	Pro	Ala	Val	37	0.314
PAV/AVI	CG	CT	GA	P/A	A/V	V/I	63	0.534
AVI/AVI	GG	TT	AA	Ala	Val	Ile	18	0.152
Total							118	

**Table 2:** Characteristics of the participants in the PROP taste testing.

	No. of Samples	Frequency	Sex/Age														Age			
			Female							Male							Min	Max	M	SD
			~20	~30	~40	~50	~60	61~	Total	~20	~30	~40	~50	~60	61~	Total				
PAV/PAV	24	0.304	1	6	3	3	1	0	14	0	2	3	1	4	0	10	20	60	37.33	11.94
PAV/AVI	43	0.544	0	5	5	9	6	1	26	0	4	3	6	3	1	17	21	65	41.74	12.00
AVI/AVI	12	0.152	0	2	1	1	2	0	6	0	0	3	2	1	0	6	22	60	41.42	12.15
Total	79	1.000	1	13	9	13	9	1	46	0	6	9	9	8	1	33	20	65	40.35	12.17

## Data Availability

The obtained sequences have been deposited in the DNA Databank of Japan (DDBJ) under accession numbers LC664752–LC664753.
